# Supplementary material for: Placental, Foetal, and Maternal Serum Metabolomic Profiles in Pregnancy-Associated Cancer: Walker-256 Tumour Model in a Time-Course Analysis
Source: Int J Mol Sci. 2023 Aug 22;24(17):13026. doi: 10.3390/ijms241713026 (PMC10487647; doi:10.3390/ijms241713026)
Supplement: Supplementary file 1 [file ijms-24-13026-s001.zip › Supplementary Figures.pdf]

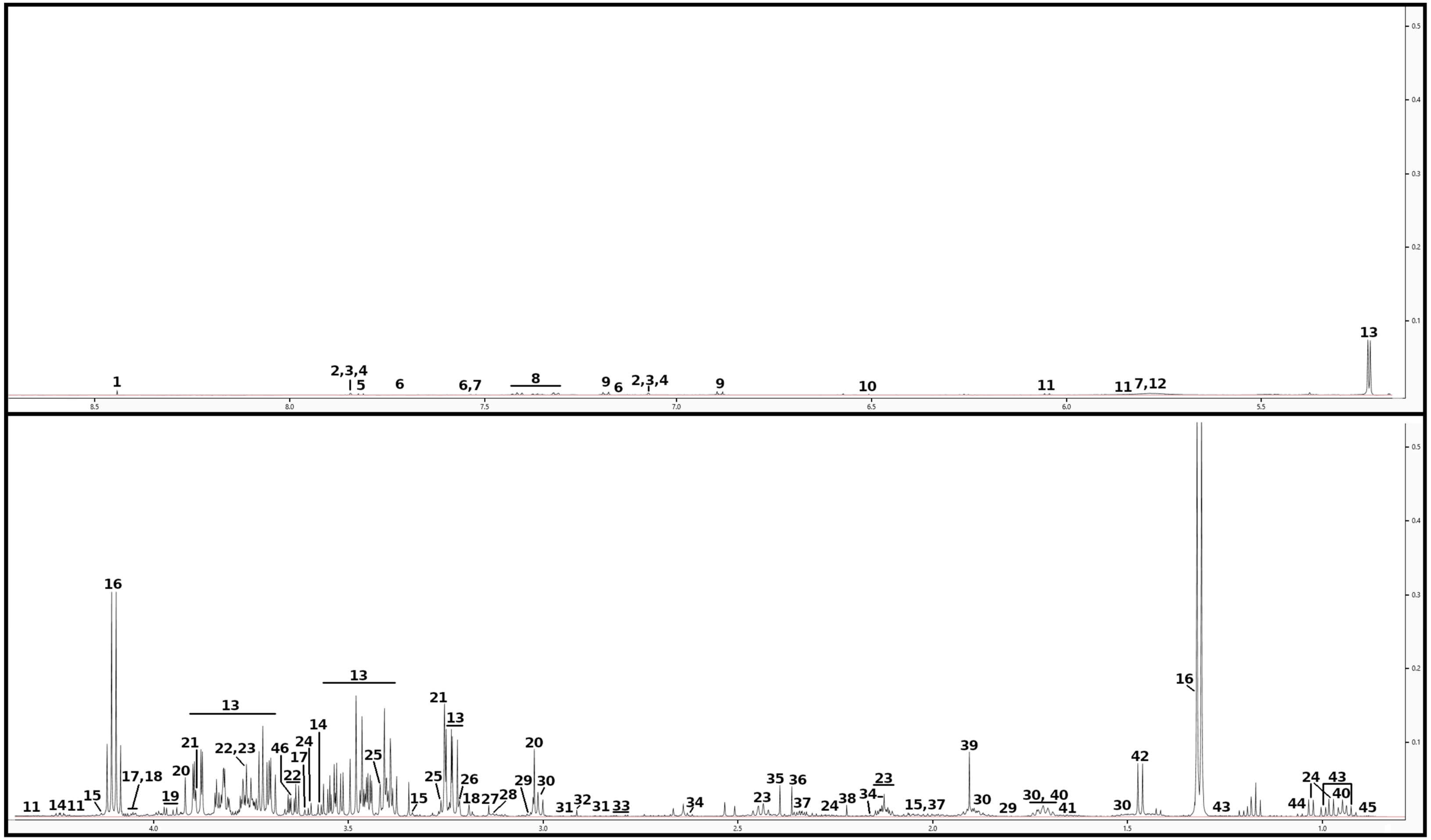

Supplementary Figure S1. Representative maternal serum ^{1}H NMR spectra of a healthy pregnant group at 12 days post-conception. The numbered peaks assigned were: 1, Formate; 2, Histamine; 3, Histidine; 4, 1-Methylhistidine; 5, Uridine; 6, Tryptophan; 7, Uracil; 8, Phenylalanine; 9, Tyrosine; 10, Fumarate; 11, Cytidine; 12, Urea; 13, lucose; 14, Threonine; 15, Proline; 16, Lactate; 17, myo-Inositol; 18, Choline; 19, Serine; 20, Creatine; 21, Betaine; 22, Glycerol; 23, Glutamine; 24, Valine; 25, Taurine; 26, ; Carnitine; 27, Dimethyl sulfone; 28, Ethanolamine; 29, Ornithine; 30, Lysine; 31, Asparagine; 32, N,N-Dimethylglycine; 33, Aspartate; 34, Methionine; 35, Succinate; 36, Pyruvate; 37, Glutamate; 38, Acetone; 39, Acetate; 40, Leucine; 41, Arginine; 42, Alanine; 43, Isoleucine; 44, Methylsuccinate; 45, Pantothenate; 46, Glycine.

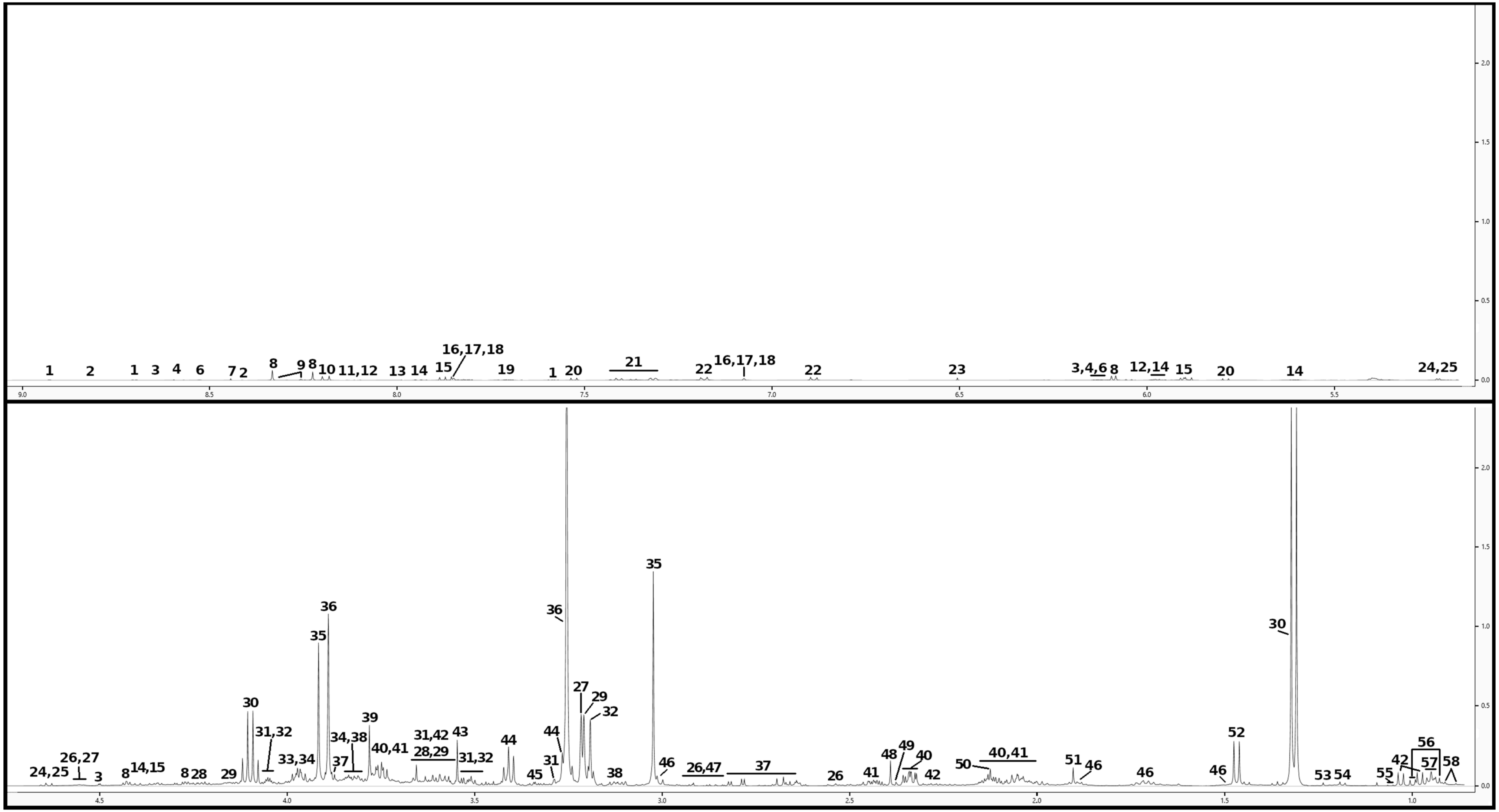

Supplementary Figure S2. Representative placenta ^{1}H NMR spectra of a healthy pregnant group at 12 days post-conception. The numbered peaks assigned were: 1,Nicotinurate; 2,NAD+; 3,AMP; 4,IMP; 5,ADP; 6,ATP; 7,Formate; 8,Inosine; 9,Adenosine; 10,Adenine; 11,GTP; 12,UMP; 13,Guanosine; 14,UDP-Glucose; 15,Uridine; 16,Histamine; 17,Histidine; 18,1-Methylhistidine; 19,Tryptophan; 20,Uracil; 21,Phenylalanine; 22,Tyrosine; 23,Fumarate; 24,Glucose; 25,Glucose-6-phosphate; 26,Glutathione; 27,Carnitine; 28,Threonine; 29,O-Phosphocholine; 30,Lactate; 31,myo-Inositol; 32,Choline; 33,O-Phosphoethanolamine; 34,Serine; 35,Creatine; 36,Betaine; 37,Aspartate; 38,Ethanolamine; 39,Guanidoacetate; 40,Glutamate; 41,Glutamine; 42,Valine; 43,Glycine; 44,Taurine; 45,Proline; 46,Lysine; 47,Asparagine; 48,Succinate; 49,Pyruvate; 50,Methionine; 51,Acetate; 52,Alanine; 53,3-Hydroxyisovalerate; 54,3-Hydroxybutyrate; 55,3-Hydroxyisobutyrate; 56,Isoleucine; 57,Leucine; 58,Pantothenate;

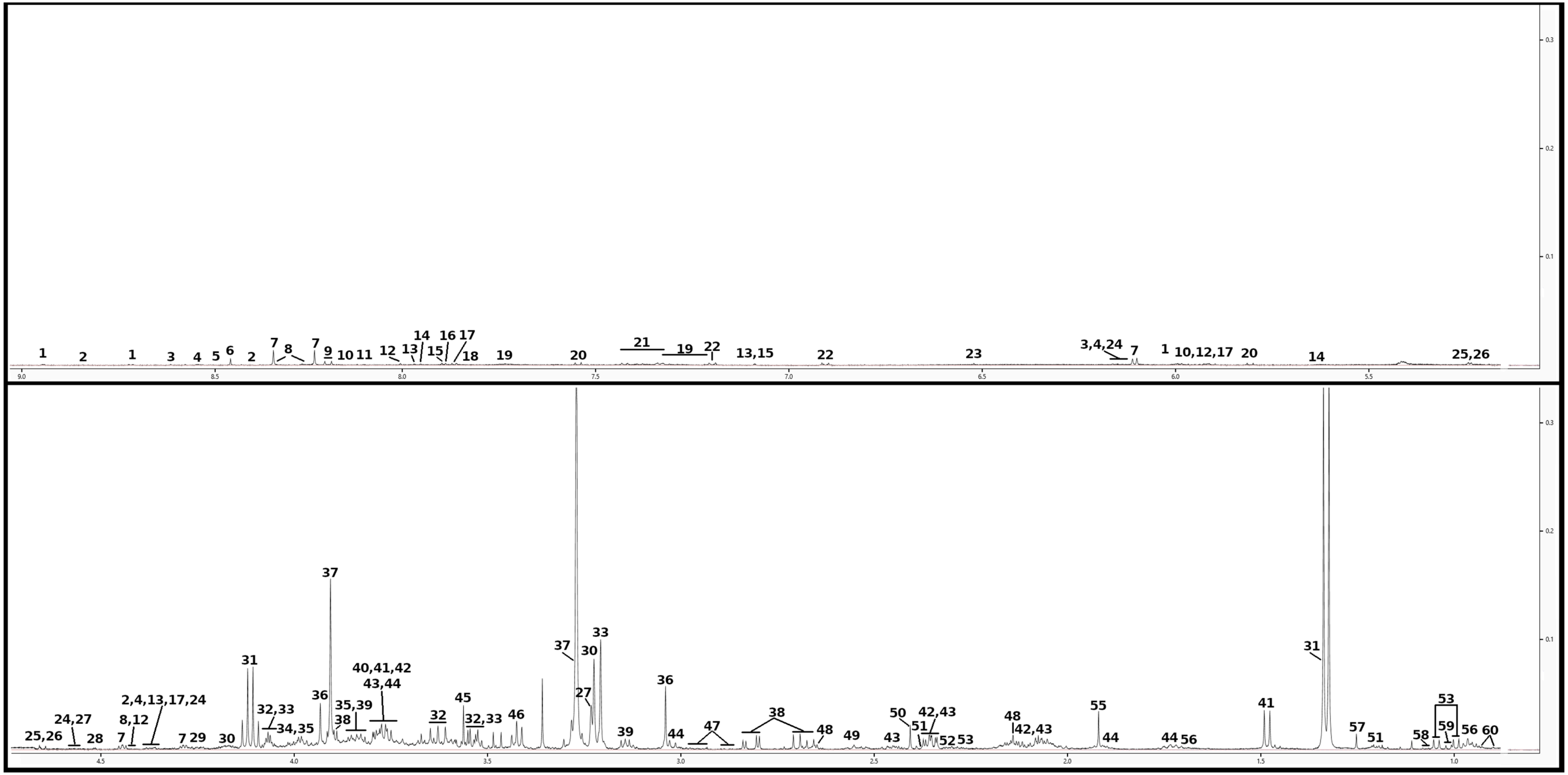

Supplementary Figure S3. Representative foetus ^{1}H NMR spectra of a healthy pregnant group at 12 days post-conception. The numbered peaks assigned were: 1,Nicotinurate; 2,NAD+; 3,AMP; 4,IMP; 5,ATP; 6,Formate; 7,Inosine; 8,Adenosine; 9,Adenine; 10,GTP; 11,UMP; 12,Guanosine; 13,1-Methylhistidine; 14,UDP-Glucose; 15,Histamine; 16,Histidine; 17,Uridine; 18,Cytidine; 19,Tryptophan; 20,Uracil; 21,Phenylalanine; 22,Tyrosine; 23,Fumarate; 24,ADP; 25,Glucose; 26,Glucose-6-phosphate; 27,Carnitine; 28,Arcorbate; 29,Threonine; 30,O-Phosphocholine; 31,Lactate; 32,Myo-Inositol; 33,Choline; 34,O-phosphoEthanolamine; 35,Serine; 36,Creatine; 37,Betaine; 38,Aspartate; 39,Ethanolamine; 40,Guanidoacetate; 41,Alanine; 42,Glutamate; 43,Glutamine; 44,Lysine; 45,Glycine; 46,Taurine; 47,Asparagine; 48,Methionine; 49,Citrate; 50,Succinate; 51,3-Hydroxybutyrate; 52,Pyruvate; 53,Valine; 54,Proline; 55,Acetate; 56,Leucine; 57,3-Hydroxyisovalerate; 58,3-Hydroxyisobutyrate; 59,Isoleucine; 60,Pantothenate.

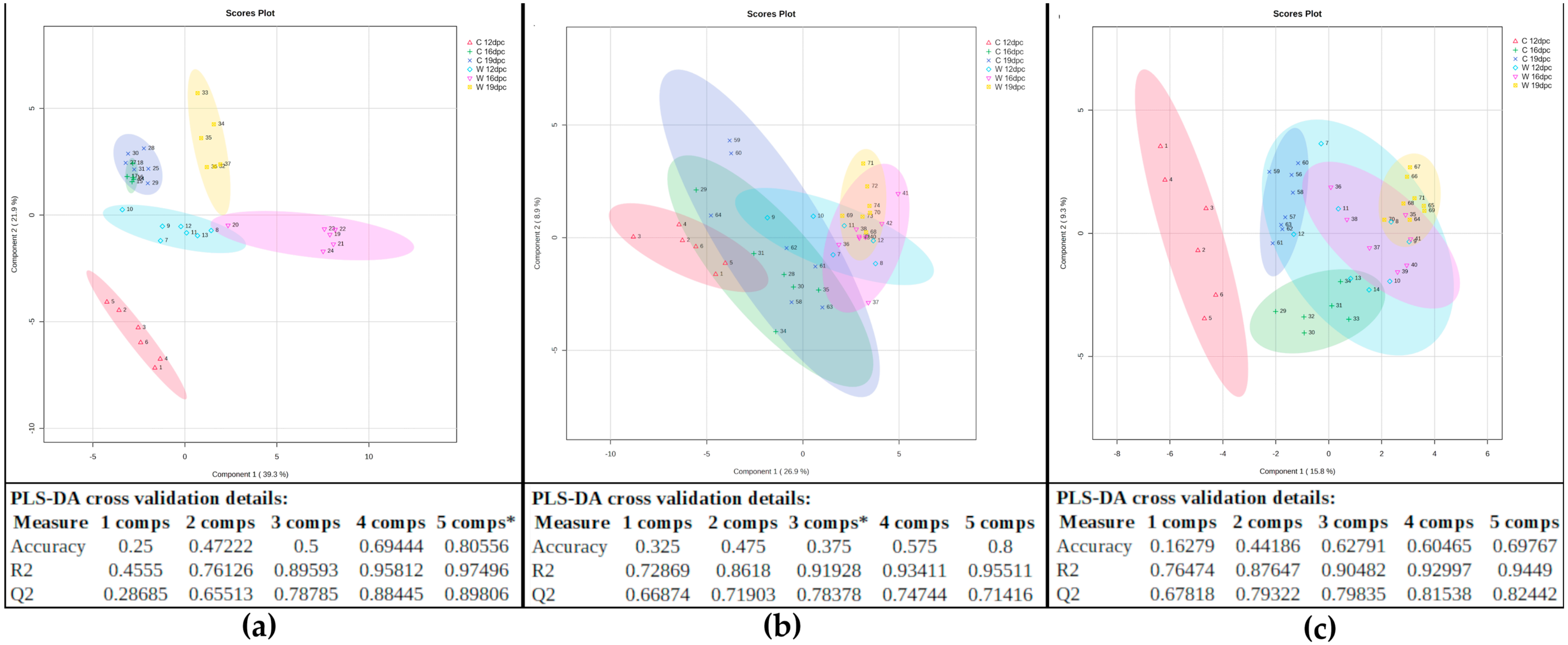

PLS-DA cross validation details:

Measure	1 comps	2 comps	3 comps	4 comps	5 comps*
Accuracy	0.25	0.47222	0.5	0.69444	0.80556
R2	0.4555	0.76126	0.89593	0.95812	0.97496
Q2	0.28685	0.65513	0.78785	0.88445	0.89806

(a)

PLS-DA cross validation details:

Measure	1 comps	2 comps	3 comps*	4 comps	5 comps
Accuracy	0.325	0.475	0.375	0.575	0.8
R2	0.72869	0.8618	0.91928	0.93411	0.95511
Q2	0.66874	0.71903	0.78378	0.74744	0.71416

(b)

PLS-DA cross validation details:

Measure	1 comps	2 comps	3 comps	4 comps	5 comps
Accuracy	0.16279	0.44186	0.62791	0.60465	0.69767
R2	0.76474	0.87647	0.90482	0.92997	0.9449
Q2	0.67818	0.79322	0.79835	0.81538	0.82442

(c)

Supplementary Figure S4. Partial Least Squares - Discriminant Analysis (PLS-DA) for experimental groups, healthy (C) and tumour-bearing (W) dams at three different gestational periods, 12-, 16- and 19- days post-conception (dpc). (**a**), maternal serum; (**b**), placenta; (**c**), foetus. The spectral data were mean-centre and divided by the standard deviation of each variable (Auto scaling), the Partial Least Squares - Discriminant Analysis (PLS-DA) models were extracted at a confidence level of 95%. The quality of fit parameters for the PLS-DA model (R2X, R2Y, and Q2Y) were then calculated and determined to validate the model. R2X and R2Y represent the fraction of the variance of the x and y variables explained by the model, respectively. Q2 is an estimate of the model's predictive ability and is calculated through cross-validation (CV). In each CV, the predicted data is compared with the original data, and the sum of squared errors is calculated. The prediction error is then summed across all samples (Predicted Residual Sum of Squares or PRESS). For convenience, PRESS is divided by the initial sum of squares and subtracted from 1 to resemble the scale of R2. Good predictions will have low PRESS or high Q2 (*). It is possible to have a negative Q2, meaning that the model is not predictive or overfitted (Szymańska et al). The online MetaboAnalyst 5.0 platform (https://www.metaboanalyst.ca/) was used to perform statistical analyses and generate the PLS-DA graphics.
