# Supplementary material for: Placental, Foetal, and Maternal Serum Metabolomic Profiles in Pregnancy-Associated Cancer: Walker-256 Tumour Model in a Time-Course Analysis
Source: Int J Mol Sci. 2023 Aug 22;24(17):13026. doi: 10.3390/ijms241713026 (PMC10487647; doi:10.3390/ijms241713026)
Supplement: Supplementary file 1 [file ijms-24-13026-s001.zip › Supplementary Table S1 - Maternal morphometric parameters.pdf]

**Supplementary Table S1**. Maternal and tumour morphometric data for experimental groups, healthy (C) and tumour-bearing (W) dams at three different gestational periods, 12-, 16- and 19-days post-conception. Data presented as mean + standard deviation. Number of animals per group, (12 dpc $n = 8$; 16 dpc $n = 6$; 19 dpc $n = 8$); W, (12 dpc $n = 10$; 16 dpc $n = 7$; 19 dpc $n = 8$). a, $p < 0.05$ compared to 12 dpc in the same group; * $p < 0.05$ compared to group C group in the same gestation period.

| | 12 dpc | 16 dpc | 19 dpc | 12 dpc | 16 dpc | 19 dpc |
|---|---|---|---|---|---|---|
| **Maternal weight (g)** | 305.80 ± 18.27 | 336.75 ± 29.35 | 333.99 ± 20.06 | 296.02 ± 40.79 | 317.81 ± 34.68 | 336.24 ± 62.44 |
| **Tumour weight (g)** | - | - | - | 16.57 ± 6.24 | 22.72 ± 5.57 | 28.86 ± 11.35a |
| **Carcass weight (g)** | 247.13 ± 20.80 | 253.53 ± 23.22 | 235.60 ± 19.19 | 204.22 ± 40.71 | 224.66 ± 38.57 | 216.59 ± 45.93 |
| **Foetal resorption** | 0.5 ± 0.75 | 0.429 ± 0.535 | 0.375 ± 0.518 | 1.5 ± 1.0* | 1.857 ± 1.574* | 0.875 ± 1.126 |
