# Supplementary material for: Placental, Foetal, and Maternal Serum Metabolomic Profiles in Pregnancy-Associated Cancer: Walker-256 Tumour Model in a Time-Course Analysis
Source: Int J Mol Sci. 2023 Aug 22;24(17):13026. doi: 10.3390/ijms241713026 (PMC10487647; doi:10.3390/ijms241713026)
Supplement: Supplementary file 1 [file ijms-24-13026-s001.zip › Supplementary Table S2 - Maternal serum metabolic profile.pdf]

**Supplementary Table S2.** Maternal serum metabolic profile for experimental groups, healthy (C) and tumour-bearing (W) pregnancy at three different gestational periods 12-, 16-, and 19-days post-conception.. Results presented as mean (mM) + standard deviation. Number of animals per group: C (12 dpc, n = 6; 16 dpc n = 5; 19 dpc n = 6); W, (12 dpc n = 7; 16 dpc n = 6; 19 dpc *n* = 6). a, $p < 0.05$ compared to group C group at 12 dpc; b, $p < 0.05$ compared to group C group at 16 dpc; * $p < 0.05$ compared to group C group in the same gestation period.

| | C | | | W | | |
|---|---|---|---|---|---|---|
| | **12 dpc** | **16 dpc** | **19 dpc** | **12 dpc** | **16 dpc** | **19 dpc** |
| 2-Aminobutyrate | 0.004 ± 0.001 | 0.003 ± 0.001 | 0.004 ± 0.001 | 0.004 ± 0.002 | 0.014 ± 0.006* | 0.009 ± 0.002* |
| Acetate | 0.119 ± 0.015 | 0.072 ± 0.002[a] | 0.061 ± 0.007[a] | 0.081 ± 0.040* | 0.053 ± 0.018 | 0.043 ± 0.014 |
| Acetone | 0.011 ± 0.002 | 0.009 ± 0.0002 | 0.007 ± 0.0002 | 0.009 ± 0.002 | 0.183 ± 0.109* | 0.017 ± 0.005 |
| Alanine | 0.262 ± 0.026 | 0.201 ± 0.003[a] | 0.199 ± 0.008[a] | 0.312 ± 0.058 | 0.514 ± 0.079* | 0.370 ± 0.023* |
| Arginine | 0.121 ± 0.028 | 0.090 ± 0.008[a] | 0.084 ± 0.014[a] | 0.092 ± 0.017* | 0.061 ± 0.027 | 0.090 ± 0.039 |
| Asparagine | 0.026 ± 0.003 | 0.020 ± 0.003[a] | 0.020 ± 0.004[a] | 0.021 ± 0.003* | 0.032 ± 0.005* | 0.029 ± 0.003* |
| Aspartate | 0.032 ± 0.003 | 0.016 ± 0.003[a] | 0.019 ± 0.004[a] | 0.023 ± 0.005* | 0.024 ± 0.004* | 0.014 ± 0.004* |
| Betaine | 0.121 ± 0.013 | 0.059 ± 0.002[a] | 0.041 ± 0.002[a] | 0.095 ± 0.010 | 0.109 ± 0.047* | 0.046 ± 0.004 |
| Carnitine | 0.017 ± 0.004 | 0.014 ± 0.0005[a] | 0.010 ± 0.001[a,b] | 0.019 ± 0.001 | 0.020 ± 0.003* | 0.013 ± 0.001* |
| Choline | 0.020 ± 0.004 | 0.013 ± 0.0005[a] | 0.012 ± 0.001[a] | 0.019 ± 0.009 | 0.029 ± 0.009* | 0.015 ± 0.003 |
| Creatine | 0.176 ± 0.042 | 0.095 ± 0.002[a] | 0.108 ± 0.013[a] | 0.197 ± 0.057 | 0.307 ± 0.083* | 0.117 ± 0.043 |
| Creatine phosphate | 0.021 ± 0.012 | 0.012 ± 0.002 | 0.018 ± 0.006 | 0.018 ± 0.005 | 0.017 ± 0.005 | 0.017 ± 0.004 |
| Creatinine | 0.014 ± 0.005 | 0.010 ± 0.001 | 0.010 ± 0.002 | 0.017 ± 0.003 | 0.033 ± 0.008 | 0.012 ± 0.002 |
| Cytidine | 0.009 ± 0.003 | 0.005 ± 0.001[a] | 0.005 ± 0.001[a] | 0.008 ± 0.002 | 0.012 ± 0.002* | 0.006 ± 0.001 |
| Dimethyl sulfone | 0.011 ± 0.001 | 0.004 ± 0.0001[a] | 0.007 ± 0.001[a,b] | 0.008 ± 0.002* | 0.005 ± 0.002 | 0.006 ± 0.001 |
| Ethanolamine | 0.038 ± 0.008 | 0.021 ± 0.002[a] | 0.024 ± 0.006[a] | 0.033 ± 0.006 | 0.043 ± 0.003* | 0.026 ± 0.008 |
| Formate | 0.027 ± 0.006 | 0.019 ± 0.0005[a] | 0.018 ± 0.001[a] | 0.028 ± 0.005 | 0.022 ± 0.007 | 0.026 ± 0.005 |
| Fumarate | 0.003 ± 0.001 | 0.001 ± 0.0002[a] | 0.002 ± 0.0001[b] | 0.003 ± 0.001 | 0.003 ± 0.0005* | 0.002 ± 0.001 |
| Glucose | 2.382 ± 0.186 | 1.485 ± 0.039[a] | 1.247 ± 0.336[a] | 1.883 ± 0.528 | 1.112 ± 0.518 | 1.527 ± 0.153 |
| Glutamate | 0.119 ± 0.017 | 0.081 ± 0.008[a] | 0.073 ± 0.010[a] | 0.111 ± 0.023 | 0.100 ± 0.018 | 0.081 ± 0.009 |
| Glutamine | 0.269 ± 0.042 | 0.204 ± 0.007[a] | 0.194 ± 0.014[a] | 0.195 ± 0.009* | 0.162 ± 0.082 | 0.208 ± 0.029 |
| Glycerol | 0.285 ± 0.070 | 0.268 ± 0.010 | 0.295 ± 0.015 | 0.305 ± 0.081 | 0.370 ± 0.096 | 0.348 ± 0.014 |
| Glycine | 0.132 ± 0.018 | 0.057 ± 0.001[a] | 0.054 ± 0.004[a] | 0.089 ± 0.013* | 0.181 ± 0.043* | 0.072 ± 0.017 |
| Guanidoacetate | 0.016 ± 0.004 | 0.013 ± 0.001 | 0.016 ± 0.003 | 0.014 ± 0.006 | 0.017 ± 0.004 | 0.017 ± 0.002 |
| Histamine | 0.008 ± 0.004 | 0.004 ± 0.001[a] | 0.005 ± 0.001[a] | 0.006 ± 0.001 | 0.009 ± 0.003* | 0.007 ± 0.003 |
| Histidine | 0.008 ± 0.002 | 0.004 ± 0.001[a] | 0.005 ± 0.001[a] | 0.005 ± 0.001* | 0.006 ± 0.002* | 0.005 ± 0.001 |
| Isoleucine | 0.051 ± 0.007 | 0.034 ± 0.001[a] | 0.030 ± 0.003[a] | 0.043 ± 0.004* | 0.057 ± 0.007* | 0.044 ± 0.010* |
| Lactate | 7.028 ± 2.324 | 3.683 ± 0.030[a] | 4.442 ± 1.145[a] | 6.541 ± 1.654 | 8.936 ± 1.838* | 5.492 ± 1.826 |
| Leucine | 0.085 ± 0.012 | 0.058 ± 0.003[a] | 0.048 ± 0.010[a] | 0.067 ± 0.008* | 0.102 ± 0.004* | 0.067 ± 0.011* |
| Lysine | 0.258 ± 0.043 | 0.212 ± 0.014 | 0.200 ± 0.029[a] | 0.246 ± 0.042 | 0.279 ± 0.063* | 0.263 ± 0.060* |
| Methionine | 0.042 ± 0.008 | 0.030 ± 0.002[a] | 0.029 ± 0.006[a] | 0.036 ± 0.006 | 0.041 ± 0.006* | 0.032 ± 0.007 |
| Methylsuccinate | 0.017 ± 0.004 | 0.015 ± 0.0005 | 0.013 ± 0.001 | 0.018 ± 0.006 | 0.034 ± 0.007* | 0.023 ± 0.002* |
| N,N-Dimethylglycine | 0.008 ± 0.002 | 0.005 ± 0.0005[a] | 0.005 ± 0.001[a] | 0.009 ± 0.001 | 0.013 ± 0.002* | 0.008 ± 0.002* |
| O-Phosphocholine | 0.001 ± 0.0005 | 0.001 ± 0.0001 | 0.002 ± 0.0001 | 0.001 ± 0.001 | 0.005 ± 0.002* | 0.002 ± 0.001 |
| Ornithine | 0.035 ± 0.016 | 0.013 ± 0.001[a] | 0.017 ± 0.005[a] | 0.033 ± 0.015 | 0.050 ± 0.012* | 0.031 ± 0.010* |
| Pantothenate | 0.004 ± 0.001 | 0.004 ± 0.0002 | 0.004 ± 0.001 | 0.005 ± 0.001 | 0.009 ± 0.003* | 0.006 ± 0.002 |
| Phenylalanine | 0.041 ± 0.005 | 0.028 ± 0.001[a] | 0.030 ± 0.002[a] | 0.040 ± 0.004 | 0.060 ± 0.009* | 0.039 ± 0.004* |
| Proline | 0.092 ± 0.013 | 0.069 ± 0.003[a] | 0.052 ± 0.011[a,b] | 0.073 ± 0.019* | 0.105 ± 0.014* | 0.076 ± 0.013* |
| Pyruvate | 0.072 ± 0.006 | 0.044 ± 0.001[a] | 0.030 ± 0.002[a] | 0.091 ± 0.038 | 0.140 ± 0.027* | 0.076 ± 0.024* |
| Serine | 0.120 ± 0.018 | 0.066 ± 0.004[a] | 0.058 ± 0.008[a] | 0.093 ± 0.008* | 0.072 ± 0.019 | 0.079 ± 0.014* |
| Succinate | 0.070 ± 0.029 | 0.037 ± 0.0005 | 0.054 ± 0.027 | 0.083 ± 0.054 | 0.143 ± 0.016 | 0.066 ± 0.042 |
| Taurine | 0.297 ± 0.037 | 0.163 ± 0.005[a] | 0.177 ± 0.043[a] | 0.223 ± 0.037* | 0.351 ± 0.085* | 0.158 ± 0.060 |
| Threonine | 0.129 ± 0.022 | 0.120 ± 0.006 | 0.117 ± 0.009 | 0.148 ± 0.025 | 0.126 ± 0.036 | 0.157 ± 0.041 |
| Tryptophan | 0.003 ± 0.001 | 0.002 ± 0.001 | 0.005 ± 0.001 | 0.014 ± 0.014* | 0.016 ± 0.006* | 0.015 ± 0.004* |
| Tyrosine | 0.050 ± 0.012 | 0.026 ± 0.002[a] | 0.023 ± 0.004[a] | 0.036 ± 0.006* | 0.068 ± 0.015* | 0.031 ± 0.005 |
| Uracil | 0.008 ± 0.002 | 0.006 ± 0.001 | 0.008 ± 0.002 | 0.015 ± 0.005* | 0.026 ± 0.004* | 0.012 ± 0.005 |
| Urea | 0.590 ± 0.095 | 0.391 ± 0.058[a] | 0.400 ± 0.040[a] | 0.525 ± 0.059 | 1.026 ± 0.223* | 0.608 ± 0.056* |
| Uridine | 0.005 ± 0.001 | 0.003 ± 0.0005[a] | 0.003 ± 0.001[a] | 0.003 ± 0.002 | 0.006 ± 0.003* | 0.003 ± 0.0001 |
| Valine | 0.105 ± 0.018 | 0.075 ± 0.001[a] | 0.064 ± 0.012[a] | 0.086 ± 0.010* | 0.119 ± 0.013* | 0.090 ± 0.019* |
| myo-Inositol | 0.078 ± 0.017 | 0.050 ± 0.004 | 0.061 ± 0.006 | 0.089 ± 0.020 | 0.167 ± 0.064* | 0.072 ± 0.010 |
| 1-Methylhistidine | 0.033 ± 0.006 | 0.021 ± 0.002[a] | 0.016 ± 0.003[a,b] | 0.023 ± 0.002* | 0.042 ± 0.005* | 0.026 ± 0.004* |
