# Supplementary material for: Placental, Foetal, and Maternal Serum Metabolomic Profiles in Pregnancy-Associated Cancer: Walker-256 Tumour Model in a Time-Course Analysis
Source: Int J Mol Sci. 2023 Aug 22;24(17):13026. doi: 10.3390/ijms241713026 (PMC10487647; doi:10.3390/ijms241713026)
Supplement: Supplementary file 1 [file ijms-24-13026-s001.zip › Supplementary Table S3 - Placental metabolic profile.pdf]

Supplementary Table S3. Placenta metabolic profile for experimental groups, healthy (C) and tumour-bearing (W) dams at three different gestational periods, 12-, 16-, and 19-days post-conception. Results presented as mean (mM) + standard deviation. Number of animals per group, C (12 dpc, $n = 6$; 16 dpc $n = 6$; 19 dpc $n = 7$); W (12 dpc $n = 6$; 16 dpc $n = 8$; 19 dpc $n = 7$). a, $p < 0.05$ compared to group C group at 12 dpc; b, $p < 0.05$ compared to group C group at 16 dpc; * $p < 0.05$ compared to group C group in the same gestation period.

	C			W		
	12 dpc	16 dpc	19 dpc	12 dpc	16 dpc	19 dpc
3-Hydroxybutyrate	0.199 ± 0.037	0.146 ± 0.092	0.110 ± 0.034	0.143 ± 0.063	0.392 ± 0.598	0.113 ± 0.036
3-Hydroxyisobutyrate	0.076 ± 0.011	0.097 ± 0.014	0.095 ± 0.011	0.077 ± 0.008	0.084 ± 0.021	0.089 ± 0.008
3-Hydroxyisovalerate	0.156 ± 0.058	0.076 ± 0.049	0.078 ± 0.062	0.031 ± 0.029	0.018 ± 0.005	0.014 ± 0.001
ADP	0.084 ± 0.022	0.085 ± 0.039	0.064 ± 0.052	0.054 ± 0.035	0.064 ± 0.038	0.055 ± 0.042
AMP	0.135 ± 0.048	0.143 ± 0.047	0.073 ± 0.052	0.101 ± 0.033	0.156 ± 0.067	0.137 ± 0.078
ATP	0.043 ± 0.014	0.046 ± 0.018	0.041 ± 0.018	0.038 ± 0.014	0.053 ± 0.017	0.055 ± 0.016
Acetate	0.876 ± 0.216	0.611 ± 0.328^{a}	0.487 ± 0.293^{a}	0.504 ± 0.124*	0.335 ± 0.230	0.323 ± 0.107*
Adenine	0.854±0.121	0.766 ± 0.177	0.822 ± 0.379	0.557 ± 0.195	0.584 ± 0.087	0.581 ± 0.109
Adenosine	0.066 ± 0.031	0.061 ± 0.024	0.052 ± 0.036^{b}	0.027 ± 0.006	0.033 ± 0.013	0.031 ± 0.013
Alanine	3.572 ± 0.214	3.412 ± 0.329	3.812 ± 0.396^{b}	3.833 ± 0.231	4.086 ± 0.457	4.380 ± 0.479*
Asparagine	0.779 ± 0.201	0.746 ± 0.176	0.740 ± 0.181	0.631 ± 0.091	0.600 ± 0.082	0.726 ± 0.096
Aspartate	3.413 ± 1.351	3.208 ± 0.960	2.417 ± 0.515	3.326 ± 1.068	2.587 ± 0.854	2.136 ± 0.822
Betaine	11.699 ± 1.719	12.209 ± 2.488	11.412 ± 2.817	10.458 ± 1.035	11.627 ± 2.669	10.432 ± 1.666
Carnitine	0.846 ± 0.116	0.811 ± 0.126	0.851 ± 0.150	0.709 ± 0.156	0.760 ± 0.131	0.873 ± 0.082
Choline	1.247 ± 0.181	1.127 ± 0.178	1.572 ± 0.440$^{a.b}$	0.985 ± 0.235	0.907 ± 0.126	0.907 ± 0.105*
Creatine	5.447 ± 1.740	2.157 ± 0.504^{a}	1.561 ± 0.568^{a}	3.784 ± 0.603*	2.562 ± 0.334	2.858 ± 0.462*
Cytidine	0.126 ± 0.058	0.074 ± 0.058^{a}	0.028 ± 0.013$^{a.b}$	0.029 ± 0.016*	0.024 ± 0.011*	0.030 ± 0.016
Ethanolamine	1.078 ± 0.227	1.130 ± 0.477	1.434 ± 0.562	0.788 ± 0.094	0.748 ± 0.104	0.850 ± 0.175
Formate	0.460 ± 0.189	0.340 ± 0.178	0.398 ± 0.381	0.486 ± 0.371	0.257 ± 0.144	0.270 ± 0.132
Fumarate	0.113 ± 0.029	0.064 ± 0.019^{a}	0.061 ± 0.025^{a}	0.045 ± 0.018*	0.045 ± 0.005*	0.044 ± 0.008
GTP	0.054 ± 0.022	0.040 ± 0.019^{a}	0.038 ± 0.020^{b}	0.024 ± 0.008*	0.031 ± 0.014	0.023 ± 0.010*
Glucose	0.917 ± 0.348	1.384 ± 0.617^{a}	1.906 ± 1.033^{a}	0.799 ± 0.371	0.695 ± 0.156*	0.779 ± 0.183*
Glucose-6-phosphate	0.921 ± 0.488	1.504 ± 0.836	1.516 ± 0.712	1.084 ± 0.974	1.033 ± 0.300	0.819 ± 0.575
Glutamate	5.590 ± 0.852	5.954 ± 1.049	6.111 ± 0.801^{a}	5.108 ± 0.537	5.918 ± 0.768	5.601 ± 0.241
Glutamine	2.156 ± 0.132	2.309 ± 0.501^{a}	2.736 ± 0.463^{a}	1.435 ± 0.220*	2.075 ± 0.289*	2.195 ± 0.277*
Glutathione	0.249 ± 0.141	0.423 ± 0.295	0.404 ± 0.319	0.117 ± 0.119	0.163 ± 0.200	0.125 ± 0.073
Glycine	2.476 ± 0.374	2.271 ± 0.397	2.247 ± 0.465	2.320 ± 0.718	2.021 ± 0.281	1.972 ± 0.358
Guanidoacetate	2.191 ± 0.820	1.246 ± 0.904^{a}	0.777 ± 0.320^{a}	1.025 ± 0.263*	0.888 ± 0.170	0.813 ± 0.184
Guanosine	0.078 ± 0.035	0.135 ± 0.050	0.125 ± 0.058	0.070 ± 0.025	0.105 ± 0.020	0.108 ± 0.039
Histamine	0.148 ± 0.140	0.090 ± 0.109^{a}	0.048 ± 0.047^{a}	0.049 ± 0.020*	0.059 ± 0.013	0.070 ± 0.033
Histidine	0.079 ± 0.069	0.047 ± 0.036	0.032 ± 0.014	0.052 ± 0.029	0.042 ± 0.010	0.032 ± 0.014
IMP	0.067 ± 0.026	0.078 ± 0.042	0.069 ± 0.030	0.053 ± 0.050	0.061 ± 0.017	0.043 ± 0.018
Inosine	0.996 ± 0.166	1.189 ± 0.222^{a}	1.130 ± 0.220^{a}	0.735 ± 0.228*	1.051 ± 0.102*	0.919 ± 0.154*
Isoleucine	0.500 ± 0.031	0.426 ± 0.051^{a}	0.421 ± 0.079$^{a.b}$	0.430 ± 0.026*	0.377 ± 0.031*	0.434 ± 0.014*
Lactate	27.101 ± 2.077	28.524 ± 3.778	28.864 ± 2.995	36.414 ± 5.930*	34.809 ± 2.025*	36.021 ± 1.580*
Leucine	1.104 ± 0.125	0.912 ± 0.233^{a}	0.913 ± 0.199^{a}	0.885 ± 0.042*	0.803 ± 0.096	0.924 ± 0.051
Lysine	1.517 ± 0.302	1.914 ± 0.299^{a}	1.866 ± 0.436^{b}	1.624 ± 0.234	1.963 ± 0.319	1.964 ± 0.256
Methionine	0.413 ± 0.016	0.332 ± 0.132	0.317 ± 0.178	0.252 ± 0.120*	0.201 ± 0.068*	0.175 ± 0.046*
NAD+	0.043 ± 0.008	0.102 ± 0.054^{a}	0.075 ± 0.067	0.064 ± 0.046	0.139 ± 0.028	0.094 ± 0.022
Nicotinurate	0.269 ± 0.056	0.227 ± 0.046^{a}	0.234 ± 0.078	0.183 ± 0.048*	0.153 ± 0.017*	0.167 ± 0.025*
O-Phosphocholine	1.150 ± 0.181	0.995 ± 0.222	1.018 ± 0.239	1.291 ± 0.224	0.941 ± 0.131	1.040 ± 0.138
O-Phosphoethanolamine	3.475 ± 0.496	3.629 ± 0.562	3.532 ± 1.050	3.165 ± 0.371	3.933 ± 0.421	3.732 ± 0.338
Pantothenate	0.169 ± 0.032	0.154 ± 0.043	0.123 ± 0.048^{a}	0.089 ± 0.024*	0.139 ± 0.022	0.155 ± 0.050*
Phenylalanine	0.554 ± 0.027	0.521 ± 0.097	0.488 ± 0.088^{a}	0.514 ± 0.027	0.419 ± 0.049*	0.476 ± 0.037
Proline	1.613 ± 0.161	1.611 ± 0.242	1.419 ± 0.157b	1.356 ± 0.396*	1.397 ± 0.161*	1.399 ± 0.187
Pyruvate	0.132 ± 0.024	0.101 ± 0.043^{a}	0.076 ± 0.020^{a}	0.078 ± 0.047*	0.079 ± 0.023	0.056 ± 0.006*
Serine	1.880 ± 0.479	2.229 ± 0.718	2.323 ± 0.477^{a}	1.409 ± 0.266	1.577 ± 0.182*	1.585 ± 0.295*
Succinate	0.575 ± 0.089	0.616 ± 0.107	0.498 ± 0.128	0.737 ± 0.194*	0.760 ± 0.098*	0.697 ± 0.079*
Taurine	5.449 ± 0.410	6.486 ± 0.610^{a}	7.363 ± 1.171^{a}	5.235 ± 0.452	6.398 ± 0.917	6.555 ± 0.895
Threonine	1.330 ± 0.222	1.578 ± 0.371^{a}	1.911 ± 0.525^{a}	1.149 ± 0.183	1.082 ± 0.259*	1.169 ± 0.102*
Tryptophan	0.215 ± 0.054	0.191 ± 0.070^{a}	0.155 ± 0.029^{a}	0.166 ± 0.031*	0.113 ± 0.020*	0.130 ± 0.029*
Tyrosine	0.517 ± 0.042	0.412 ± 0.078^{a}	0.411 ± 0.121^{a}	0.432 ± 0.072*	0.303 ± 0.047*	0.376 ± 0.064
UDP-glucose	0.210 ± 0.062	0.284 ± 0.121	0.198 ± 0.141	0.252 ± 0.060	0.276 ± 0.055	0.257 ± 0.066
UMP	0.153 ± 0.048	0.138 ± 0.077	0.097 ± 0.077	0.087 ± 0.016	0.126 ± 0.041	0.085 ± 0.023
Uracil	0.451 ± 0.096	0.400 ± 0.115	0.466 ± 0.254	0.451 ± 0.113	0.353 ± 0.059	0.353 ± 0.024
Uridine	0.658 ± 0.093	0.441 ± 0.191^{a}	0.353 ± 0.093^{a}	0.292 ± 0.159*	0.264 ± 0.075	0.285 ± 0.073
Valine	1.078 ± 0.130	0.906 ± 0.120^{a}	0.902 ± 0.138^{a}	0.863 ± 0.055*	0.746 ± 0.103*	0.855 ± 0.046
myo-Inositol	1.986 ± 0.652	2.183 ± 0.494	2.033 ± 0.438	2.589 ± 0.329	2.341 ± 0.298	2.347 ± 0.123
1-Methylhistidine	0.181 ± 0.030	0.259 ± 0.104^{a}	0.210 ± 0.047^{b}	0.302 ± 0.022*	0.224 ± 0.029*	0.234 ± 0.033*
