# Supplementary material for: Placental, Foetal, and Maternal Serum Metabolomic Profiles in Pregnancy-Associated Cancer: Walker-256 Tumour Model in a Time-Course Analysis
Source: Int J Mol Sci. 2023 Aug 22;24(17):13026. doi: 10.3390/ijms241713026 (PMC10487647; doi:10.3390/ijms241713026)
Supplement: Supplementary file 1 [file ijms-24-13026-s001.zip › Supplementary Table S4 - Foetal metabolic profile.pdf]

Supplementary Table S4. Foetus metabolic profile for experimental groups, healthy (C) and tumour-bearing (W) pregnancy at three different gestational periods 12-, 16-, and 19-days post-conception.. Results presented as mean (mM) + standard deviation. Number of animals per group: C (12 dpc, $n = 6$; 16 dpc $n = 6$; 19 dpc $n = 8$); W (12 dpc $n = 7$; 16 dpc $n = 7$; 19 dpc n = 8). a, $p < 0.05$ compared to group C group at 12 dpc; b, $p < 0.05$ compared to group C group at 16 dpc; * $p < 0.05$ compared to group C group in the same gestation period.

	C			W		
	**12 dpc**	**16 dpc**	**19 dpc**	**12 dpc**	**16 dpc**	**19 dpc**
3-Hydroxybutyrate	0.231 ± 0.077	0.136 ± 0.036	0.127 ± 0.029	0.163 ± 0.080	0.404 ± 0.674	0.700 ± 1.410
3-Hydroxyisobutyrate	0.112 ± 0.022	0.127 ± 0.023	0.089 ± 0.043	0.135 ± 0.008	0.104 ± 0.029	0.110 ± 0.016
3-Hydroxyisovalerate	0.211 ± 0.217	0.036 ± 0.055	0.046 ± 0.042	0.021 ± 0.024	0.046 ± 0.067	0.012 ± 0.003
ADP	0.238 ± 0.061	0.073 ± 0.030[a]	0.141 ± 0.059[a]	0.185 ± 0.074	0.157 ± 0.059*	0.209 ± 0.060*
AMP	0.516 ± 0.194	0.645 ± 0.140	0.224 ± 0.110[a.b]	0.581 ± 0.173	0.575 ± 0.066	0.470 ± 0.271*
ATP	0.073 ± 0.021	0.064 ± 0.012	0.097 ± 0.022[a.b]	0.099 ± 0.034*	0.059 ± 0.006	0.130 ± 0.020*
Acetate	1.080 ± 1.113	0.407 ± 0.338	0.523 ± 0.328	0.326 ± 0.185	0.290 ± 0.271	0.294 ± 0.219
Adenine	0.652 ± 0.420	0.266 ± 0.058[a]	0.607 ± 0.221[b]	0.305 ± 0.124*	0.324 ± 0.115	0.322 ± 0.076*
Adenosine	0.057 ± 0.040	0.030 ± 0.007[a]	0.030 ± 0.012[a]	0.020 ± 0.012*	0.035 ± 0.008	0.022 ± 0.006
Alanine	4.654 ± 1.386	5.285 ± 0.439	4.827 ± 1.192	6.848 ± 1.423*	6.193 ± 0.713	7.243 ± 1.044*
Asparagine	0.626 ± 0.365	1.008 ± 0.224	0.812 ± 0.206	1.035 ± 0.136	0.811 ± 0.117	0.855 ± 0.170
Aspartate	1.666 ± 1.584	1.280 ± 0.418	1.295 ± 0.524	1.176 ± 0.281	1.035 ± 0.391	1.036 ± 0.243
Betaine	2.598 ± 4.073	0.456 ± 0.171	0.455 ± 0.093	0.542 ± 0.110	0.446 ± 0.159	0.362 ± 0.075
Carnitine	0.369 ± 0.092	0.326 ± 0.072	0.299 ± 0.116[a]	0.362 ± 0.083	0.270 ± 0.035*	0.267 ± 0.054
Choline	0.771 ± 0.131	0.552 ± 0.119	0.549 ± 0.132[a]	0.559 ± 0.137*	0.470 ± 0.092*	0.403 ± 0.065*
Citrate	0.458 ± 0.324	0.263 ± 0.091	0.282 ± 0.122	0.284 ± 0.169	0.273 ± 0.110	0.235 ± 0.090
Creatine	1.944 ± 0.221	3.074 ± 1.182	8.306 ± 6.598[a.b]	2.435 ± 0.365	2.983 ± 0.235	3.726 ± 1.257*
Cytidine	0.125 ± 0.043	0.084 ± 0.013	0.097 ± 0.037	0.123 ± 0.022	0.093 ± 0.017	0.106 ± 0.065
Ethanolamine	1.427 ± 0.451	0.659 ± 0.130[a]	0.545 ± 0.292[a]	0.540 ± 0.259*	0.655 ± 0.176	0.686 ± 0.602
Formate	0.746 ± 0.664	0.282 ± 0.320	0.310 ± 0.222	0.321 ± 0.294	0.190 ± 0.174	0.298 ± 0.213
Fumarate	0.084 ± 0.027	0.054 ± 0.009[a]	0.084 ± 0.018[a.b]	0.066 ± 0.015*	0.062 ± 0.019	0.072 ± 0.020
GTP	0.077 ± 0.025	0.034 ± 0.010[a]	0.037 ± 0.013[a]	0.039 ± 0.012*	0.028 ± 0.014	0.029 ± 0.017
Glucose	0.519 ± 0.223	1.439 ± 0.461[a]	1.305 ± 0.760[a]	0.263 ± 0.163	0.766 ± 0.316*	0.678 ± 0.334*
Glucose-6-phosphate	0.658 ± 0.364	0.758 ± 0.253	1.249 ± 0.377[a.b]	0.421 ± 0.422	0.690 ± 0.294	0.541 ± 0.357*
Glutamate	5.496 ± 2.771	7.926 ± 2.059	7.379 ± 2.818	7.748 ± 0.523	8.259 ± 1.673	8.810 ± 1.324
Glutamine	1.992 ± 0.901	3.504 ± 0.242[a]	3.638 ± 0.511[a]	2.684 ± 0.375*	3.508 ± 0.721	3.306 ± 0.399
Glutathione	0.108 ± 0.094	0.105 ± 0.054	0.202 ± 0.097	0.154 ± 0.179	0.162 ± 0.212	0.120 ± 0.111
Glycine	1.988 ± 0.623	2.728 ± 0.416	2.178 ± 0.361	2.476 ± 0.470	3.029 ± 0.584	2.670 ± 1.127
Guanidoacetate	0.362 ± 0.271	0.239 ± 0.055	0.474 ± 0.136	0.236 ± 0.132	0.206 ± 0.062	0.224 ± 0.153
Guanosine	0.044 ± 0.013	0.021 ± 0.003	0.039 ± 0.019[b]	0.021 ± 0.006*	0.017 ± 0.005	0.018 ± 0.007*
Histamine	0.086 ± 0.050	0.074 ± 0.011	0.141 ± 0.155	0.052 ± 0.021	0.065 ± 0.034	0.075 ± 0.056
Histidine	0.082 ± 0.048	0.092 ± 0.054	0.389 ± 0.528	0.050 ± 0.016	0.048 ± 0.016	0.039 ± 0.016
IMP	0.173 ± 0.058	0.213 ± 0.130	0.557 ± 0.187[a.b]	0.076 ± 0.051*	0.108 ± 0. 40	0.050 ± 0.022*
Inosine	0.486 ± 0.441	0.281 ± 0.072	0.501 ± 0.193	0.168 ± 0.111	0.299 ± 0.185*	0.254 ± 0.090
Isoleucine	0.345 ± 0.095	0.500 ± 0.138[a]	0.400 ± 0.139[b]	0.414 ± 0.098	0.401 ± 0.072	0.466 ± 0.123
Lactate	28.227 ± 5.153	31.577 ± 4.598[a]	26.380 ± 2.494[b]	33.349 ± 3.260*	31.703 ± 2.605	35.092 ± 2.610*
Leucine	0.732 ± 0.199	0.966 ± 0.280	0.852 ± 0.306	0.841 ± 0.261	0.778 ± 0.136	0.893 ± 0.206
Lysine	1.571 ± 0.307	2.165 ± 0.509[a]	1.739 ± 0.579[b]	1.994 ± 0.240*	1.807 ± 0.218*	1.718 ± 0.424
Methionine	0.298 ± 0.118	0.261 ± 0.194	0.363 ± 0.185	0.314 ± 0.217	0.210 ± 0.168	0.183 ± 0.093
NAD+	0.049 ± 0.019	0.095 ± 0.038	0.035 ± 0.042[b]	0.123 ± 0.080*	0.083 ± 0.044	0.080 ± 0.031
Nicotinurate	0.207 ± 0.077	0.134 ± 0.020[a]	0.188 ± 0.041[b]	0.124 ± 0.055*	0.125 ± 0.041	0.098 ± 0.028*
O-Phosphocholine	2.755 ± 1.301	1.830 ± 0.194[a]	1.460 ± 0.435[a]	3.117 ± 0.701	1.970 ± 0.487	1.419 ± 0.423
O-Phosphoethanolamine	2.551 ± 0.955	3.392 ± 1.139	2.484 ± 0.435	3.705 ± 0.976	3.893 ± 0.893	2.433 ± 0.676
Pantothenate	0.179 ± 0.066	0.404 ± 0.189[a]	0.317 ± 0.186[a.b]	0.194 ± 0.082	0.348 ± 0.076	0.310 ± 0.063
Phenylalanine	0.392 ± 0.207	0.463 ± 0.143	0.497 ± 0.219	0.491 ± 0.167	0.425 ± 0.136	0.571 ± 0.119
Proline	1.793 ± 0.509	1.526 ± 0.517	1.427 ± 0.385[a]	1.854 ± 0.464	1.424 ± 0.316*	1.408 ± 0.473
Pyruvate	0.113 ± 0.038	0.093 ± 0.019[a]	0.101 ± 0.014[a]	0.101 ± 0.028*	0.074 ± 0.015	0.084 ± 0.009
Serine	1.663 ± 0.322	1.984 ± 0.096	2.114 ± 0.868	1.698 ± 0.213	1.780 ± 0.511	1.700 ± 0.239
Succinate	0.767 ± 0.297	0.931 ± 0.267	0.555 ± 0.155[b]	0.935 ± 0.277	0.950 ± 0.207	0.969 ± 0.168*
Taurine	7.739 ± 3.587	9.408 ± 2.660	10.334 ± 2.911	8.942 ± 2.165	9.059 ± 1.287	7.922 ± 1.400
Threonine	1.292 ± 0.281	1.497 ± 0.082	1.674 ± 0.708	1.470 ± 0.392	1.681 ± 0.414	1.445 ± 0.199
Tryptophan	0.174 ± 0.067	0.109 ± 0.049	0.142 ± 0.092	0.114 ± 0.070	0.098 ± 0.070	0.133 ± 0.081
Tyrosine	0.331 ± 0.219	0.366 ± 0.135	0.422 ± 0.221	0.381 ± 0.162	0.331 ± 0.093	0.463 ± 0.136
UDP-glucose	0.194 ± 0.088	0.247 ± 0.097	0.259 ± 0.090[a]	0.303 ± 0.028*	0.315 ± 0.045*	0.320 ± 0.038*
UMP	0.367 ± 0.292	0.439 ± 0.135	0.335 ± 0.142	0.366 ± 0.175	0.360 ± 0.103	0.325 ± 0.172
Uracil	0.194 ± 0.149	0.128 ± 0.047	0.072 ± 0.033	0.204 ± 0.124	0.123 ± 0.019	0.187 ± 0.192
Uridine	0.225 ± 0.101	0.234 ± 0.071	0.363 ± 0.261	0.186 ± 0.086	0.286 ± 0.056*	0.252 ± 0.084
Valine	0.883 ± 0.360	1.137 ± 0.530[a]	0.958 ± 0.339[b]	1.014 ± 0.230	0.806 ± 0.143	0.999 ± 0.170
myo-Inositol	2.655 ± 0.887	2.908 ± 0.890	2.839 ± 1.244	3.018 ± 0.556	3.840 ± 0.507	3.368 ± 0.823
1-Methylhistidine	0.233 ± 0.057	0.292 ± 0.250[a]	0.232 ± 0.114[b]	0.288 ± 0.119	0.180 ± 0.094*	0.213 ± 0.043
