# Supplementary material for: Placental, Foetal, and Maternal Serum Metabolomic Profiles in Pregnancy-Associated Cancer: Walker-256 Tumour Model in a Time-Course Analysis
Source: Int J Mol Sci. 2023 Aug 22;24(17):13026. doi: 10.3390/ijms241713026 (PMC10487647; doi:10.3390/ijms241713026)
Supplement: Supplementary file 1 [file ijms-24-13026-s001.zip › Supplementary Table S5 - A time course metabolic pathway - Serum, placenta and foetus.pdf]

Supplementary Table S5. A time-course metabolites pathways along the health pregnacy at three different stages.

Legend: Health (C) group. Serum, Placenta, and Foetus impacted metabolic pathways. ⇑, enhanced; ⇓ decreased; —, unchanged metabolite. *, $p < 0.05$; **, $p < 0.01$; ***, $p < 0.001$; ****, $p < 0.0001$.

		C								
		Serum			Placenta			Foetus		
		16 *VS* 12 **(dpc)**	19 *VS* 12 **(dpc)**	19 *VS* 16 **(dpc)**	16 *VS* 12 **(dpc)**	19 *VS* 12 **(dpc)**	19 *VS* 16 **(dpc)**	16 *VS* 12 **(dpc)**	19 *VS* 12 **(dpc)**	19 *VS* 16 **(dpc)**
**Genetic Processing - Translation**	Aminoacyl-tRNA biosynthesis									
	P value (FDR)	****	****	*	****	****	-	-	-	-
	Alanine	⇓	⇓	–	–	–	–	–	–	–
	Arginine	⇓	⇓	–	–	–	–	–	–	–
	Asparagine	⇓	⇓	–	–	–	–	–	–	–
	Aspartate	⇓	⇓	–	–	–	–	–	–	–
	Glutamate	⇓	⇓	–	–	–	–	–	–	–
	Glutamine	⇓	⇓	–	–	–	–	–	–	–
	Glycine	⇓	⇓	–	–	–	–	–	–	–
	Histidine	⇓	⇓	–	–	–	–	–	–	–
	Isoleucine	⇓	⇓	–	⇓	⇓	–	–	–	–
	Leucine	–	⇓	–	–	–	–	–	–	–
	Lysine	–	⇓	–	⇑	–	–	–	–	–
	Methionine	⇓	⇓	–	–	–	–	–	–	–
	Phenylalanine	⇓	⇓	–	–	–	–	–	–	–
	Proline	⇓	⇓	⇓	–	–	–	–	–	–
	Serine	–	–	–	–	–	–	–	–	–
	Threonine	–	–		–	–	–	–	–	–
	Tryptophan	–	–	–	–	⇓	–	–	–	–
	Tyrosine	⇓	⇓	–	⇓	⇓	–	–	–	–
	Valine	⇓	⇓	–	⇓	⇓	–	–	–	–
**Amino acid metabolism Amino acid metabolism**	Alanine, aspartate and glutamate metabolism									
	P value (FDR)	****	****	>0.05	*	**	-	**	*	*
	Alanine	⇓	⇓	–	–	–	–	–	–	–
	Asparagine	⇓	⇓	–	–	–	–	–	–	–
	Aspartate	⇓	⇓	–	–	–	–	–	–	–
	Fumarate	⇓	–	⇑	⇓	⇓	–	⇓	–	⇑
	Glutamate	⇓	⇓	–	–	–	–	–	–	–
	Glutamine	⇓	⇓	–	–	⇑	–	⇑	⇑	–
	Pyruvate	⇓	⇓	–	⇓	⇓	–	⇓	⇓	⇓
	Succinate	–	–	–	–	–	–	–	–	–
	Arginine biosynthesis									
	P value (FDR)	****	****	>0.05	-	*	-	**	-	-
	Arginine	⇓	⇓	–	–	–	–	–	–	–
	Aspartate	⇓	⇓	–	–	–	–	–	–	–
	Fumarate	⇓	–	–	–	⇓	–	⇓	–	–
	Glutamate	⇓	⇓	–	–	–	–	–	–	–
	Glutamine	⇓	⇓	⇑	–	⇑	–	⇑	–	–
	Ornithine	⇓	⇓	–	–	–	–	–	–	–
	Urea	⇓	⇓	–	–	–	–	–	–	–
	Histidine metabolism									
	P value (FDR)	****	****	>0.05	*	-	>0.05	-	-	-
	1-Mehyl-histidine	⇓	⇓	⇓	⇑	–	⇓	–	–	–
	Aspartate	⇓	⇓	–	–	–	–	–	–	–
	Glutamine	⇓	⇓	–	–	–	–	–	–	–
	Histamine	⇓	⇓	–	–	–	–	–	–	–
	Histidine	⇓	⇓	–	⇓	–	–	–	–	–
	Glycine, serine and threonine metabolism									
	P value (FDR)	****	****	-	**	****	-	*	**	-
	Betaine	⇓	⇓	–	–	–	–	–	–	–
	Choline	⇓	⇓	–	–	–	–	⇓	⇓	–
	Creatine	⇓	⇓	–	⇓	⇓	–	–	⇑	–
	Glutamine	–	–	–	–	⇑	–	–	–	–
	Glycine	⇓	⇓	–	–	–	–	–	–	–
	Guanidoacetate	–	–	–	⇓	⇓	–	–	–	–
	N,N-DG	⇓	⇓	–	–	–	–	–	–	–
	Pyruvate	⇓	⇓	–	⇓	⇓	–	⇑	⇓	–
	Serine	–	–	–	–	–	–	–	–	–
	Threonine	–	–	–	–	⇑	–	–	–	–
	Arginine and proline metabolism									
	P value (FDR)	**	**	>0.05	*	**	-	-	*	-
	Arginine	⇓	⇓	–	–	–	–	–	–	–
	Creatine	⇓	⇓	–	⇓	⇓	–	–	⇑	–
	Glutamine	⇓	⇓	–	–	–	–	–	–	–
	Guanidoacetate	–	–	–	⇓	⇓	–	–	–	–
	Ornitine	⇓	⇓	–	–	–	–	–	–	–
	Proline	⇓	⇓	⇓	–	–	–	–	–	–
	Pyruvate	⇓	⇓	–	⇓	⇓	–	–	⇓	–
	Valine, leucine and isoleucine biosynthesis									
	P value (FDR)	**	***	>0.05	**	****	-	-	-	-
	Isoleucine	⇓	⇓	–	⇓	⇓	–	–	–	–
	Leucine	⇓	⇓	–	–	–	–	–	–	–
	Proline	–	–	⇓	–	–	–	–	–	–
	Threonine	–	–	–	–	–	–	–	–	–
	Valine	⇓	⇓	–	⇓	⇓	–	–	–	–
	Phenylalanine, Tyrosine and Tryptophan biosynthesis									
	P value (FDR)	*	*	-	*	*	-	-	-	-
	Phenylalanine	⇓	⇓	–	–	–	–	–	–	–
	Tyrosine	⇓	⇓	–	⇓	⇓	–	–	–	–
**Carbohydrate metabolism**	Glyoxylate and dicarboxylate metabolism									
	P value (FDR)	****	****	-	-	**	-	*	*	-
	Acetate	⇓	⇓	–	–	⇓	–	–	–	–
	Formate	⇓	⇓	–	–	–	–	–	–	–
	Glutamate	⇓	⇓	–	–	–	–	–	–	–
	Glutamine	⇓	⇓	–	–	⇑	–	⇑	⇑	–
	Glycine	⇓	⇓	–	–	–	–	–	–	–
	Pyruvate	⇓	⇓	–	–	⇓	–	⇓	⇓	–
	Serine	–	–	–	–	–	–	–	–	–
	Uracil	–	–	–	–	–	–	–	–	–
	Valine	–	–	–	–	–	–	–	–	–
	Pyruvate metabolism									
	P value (FDR)	**	-	>0.05	**	**	-	*	-	*
	Acetate	⇓	–	–	⇓	⇓	–	–	–	–
	Fumarate	⇓	–	⇑	⇓	⇓	–	⇓	–	⇑
	Lactate	⇓	–	–	–	–	–	–	–	⇓
	Pyruvate	⇓	–	–	⇓	⇓	–	⇓	–	–
	Glycolysis / Gluconeogenesis									
	P value (FDR)	*	*	-	*	**	-	*	*	-
	Acetate	⇓	⇓	–	⇓	⇓	–	–	–	–
	Glucose	⇓	⇓	–	⇑	⇑	–	⇓	⇑	–
	G-6-P	–	–	–	–	–	–	–	⇑	–
	Lactate	⇓	⇓	–	–	–	–	–	–	–
	Pyruvate	⇓	⇓	–	⇓	⇓	–	⇓	⇓	–
	Citrate cycle (TCA cycle)									
	P value (FDR)	*	-	-	*	*	-	*	-	*
	Fumarate	⇑	–	–	⇓	⇓	–	⇓	–	⇑
	Pyruvate	–	–	–	⇓	⇓	–	⇓	–	–
	Succinate	–	–	–	–	–	–	–	–	⇓
**Metabolism of cofactors and vitamins**	Pantothenate and CoA biosynthesis									
	P value (FDR)	-	-	-	-	*	-	-	-	-
	Aspartate	–	–	–	–	–	–	–	–	–
	Pantothenate	–	–	–	–	⇓	–	–	–	–
	Uracil	–	–	–	–	–	–	–	–	–
	Valine	–	–	–	–	⇓	–	–	–	–
**Nucleotide metabolism**	Purine metabolism									
	P value (FDR)	-	-	-	-	-	-	****	****	****
	Adenine	–	–	–	–	–	–	⇓	⇓	⇑
	Adenosine	–	–	–	–	–	–	⇓	–	–
	ADP	–	–	–	–	–	–	⇓	⇓	⇑
	AMP	–	–	–	–	–	–	–	⇓	⇓
	ATP	–	–	–	–	–	–	–	–	⇑
	Glutamine	–	–	–	–	–	–	⇑	⇑	–
	GTP	–	–	–	–	–	–	⇓	⇓	–
	Guanosine	–	–	–	–	–	–	⇓	–	⇑
	IMP	–	–	–	–	–	–	–	⇑	⇑
	Inosine	–	–	–	–	–	–	–	–	⇑
